# Potential impact of antimicrobial resistance in wildlife, environment and human health

**DOI:** 10.3389/fmicb.2014.00023

**Published:** 2014-02-05

**Authors:** Hajer Radhouani, Nuno Silva, Patrícia Poeta, Carmen Torres, Susana Correia, Gilberto Igrejas

**Affiliations:** ^1^Institute for Biotechnology and Bioengineering, Centre of Genomics and Biotechnology, University of Trás-os-Montes and Alto DouroVila Real, Portugal; ^2^Department of Genetics and Biotechnology, University of Trás-os-Montes and Alto DouroVila Real, Portugal; ^3^Animal and Veterinary Research Centre, University of Trás-os-Montes and Alto DouroVila Real, Portugal; ^4^Veterinary Science Department, University of Trás-os-Montes and Alto DouroVila Real, Portugal; ^5^Biochemistry and Molecular Biology Area, University of La RiojaLogroño, Spain

**Keywords:** antimicrobial resistance, *Escherichia coli*, enterococci, phylogenetic groups, wild animals, virulence factors

## Abstract

Given the significant spatial and temporal heterogeneity in antimicrobial resistance distribution and the factors that affect its evolution, dissemination, and persistence, it is important to highlight that antimicrobial resistance must be viewed as an ecological problem. Monitoring the resistance prevalence of indicator bacteria such as *Escherichia coli* and enterococci in wild animals makes it possible to show that wildlife has the potential to serve as an environmental reservoir and melting pot of bacterial resistance. These researchers address the issue of antimicrobial-resistant microorganism proliferation in the environment and the related potential human health and environmental impact.

## INTRODUCTION

Microorganisms play an important role in the cycling of elements at a global scale, thus profoundly and directly affecting the environments, in which all of life evolves. While microorganisms affect the environment, the environment in turn also engenders evolutionary pressures on the microorganisms themselves (Reid and Buckley, 2011).

Recent advances in DNA sequencing, high-throughput technologies, and genetic manipulation systems have permitted empirical studies that directly characterize the molecular and genomic bases of evolution (Wagner, 2008; Conrad et al., 2011). This launched the challenge of unraveling the genotype-phenotype connection, with implications not only for the investigation of evolution, but also physiology, disease risk, development, and biodiversity (Reid and Buckley, 2011).

In microbial populations, evolutionary change is supplied by two sources of new variation: horizontal transfer of genetic material from other, sometimes distantly related species, and mutation. Though, in the short run, mutation is the primary source of new genetic variation driving evolutionary change in microbial populations. In an asexual microbial species, evolutionary changes will happen by the repeated selection of new clones carrying adaptively favorable mutations (Adams, 2004).

In fact, firstly, genomic sequencing can determine the complete set of mutations responsible for an advanced phenotype, and has led to the discovery that interactions between these mutations are very usual. Secondly, adaptive mutations commonly target regulatory mechanisms. Thirdly, principles of systems-level optimization cause the genetic changes seen in adaptive evolution, and with a systems-level understanding, these optimization principles can be harnessed for the purposes of metabolic engineering. Fourthly, mutant sub-populations of enhanced fitness invariably arise in growing populations, but their dynamics in the population are complicated due to factors such as natural selection, clonal interference, drift, and frequency-dependent selection (Barrick et al., 2009).

Antibiotic-resistant bacteria are extremely important to human health, but the wild reservoirs of resistance determinants are poorly understood. The origins of antimicrobial resistance in the wildlife is important to human health because of the increasing importance of zoonotic diseases as well as the need for predicting emerging resistant pathogens. Wild animals provide a biological mechanism for the spread of antibiotic resistance genes. Antimicrobial-resistant *Escherichia coli and Enterococcus*spp. isolates originating from wildlife species were reported for the first time from Japanese wild birds (Sato et al., 1978). With the new millennium several studies, in different continents, have described the occurrence of antimicrobial-resistant in these bacteria species in wildlife (Souza et al., 1999; Sherley et al., 2000; Gakuya et al., 2001; Lillehaug et al., 2005; Skurnik et al., 2006; Dolejska et al., 2007; Gaukler et al., 2009; Schierack et al., 2009; Allen et al., 2010; Silva et al., 2010; Marinho et al., 2013; Navarro-Gonzalez et al., 2013; Pesapane et al., 2013; Santos et al., 2013). On the other hand, antimicrobial resistance have been described in others important pathogens, such as *Salmonella* spp. (Botti et al., 2013) and methicillin-resistant *Staphylococcus aureus* (MRSA; Porrero et al., 2013) in wild animals.

Proximity to human activities influences the antibiotic resistance profiles of the gut bacteria of wild mammals, which live in densely populated microbial habitats in which antibiotics select for resistance. While various bacterial species are important in terms of multiresistance and nosocomial infections in human and veterinary medicine, we consider the Gram-positive vancomycin-resistant enterococci (VRE) and Extended-spectrum Beta-Lactamases producing Gram-negative bacteria like *E. coli* (ESBL-*E. coli*) as being key indicator pathogens to trace the evolution of multiresistant bacteria in the environment and wildlife.

*Enterococcus* spp. are Gram-positive facultative anaerobic bacteria, spherical, which occur singly, in pairs or short chains and fit within the general definition of lactic acid bacteria (Ciftci et al., 2009). Most enterococci are not virulent and are considered relatively harmless, with little potential for human infection. However, they have also been identified as nosocomial opportunistic pathogens with increased resistance to antimicrobial approved agents (Chenoweth and Schaberg, 1990). Incidence of VRE among wild animals has been reported in several countries (Mallon et al., 2002; Figueiredo et al., 2009; Ishihara et al., 2013), including in remote areas (Silva et al., 2011a).

*Escherichia coli* is the head of the large bacterial family, *Enterobacteriaceae*, the enteric bacteria, which are facultatively anaerobic Gram-negative (Sorum and Sunde, 2001). This intestinal bacterium can be easily disseminated in different ecosystems. For this reason, fecal *E. coli* is considered to be an important indicator for the selective pressure exerted by the use of antimicrobials on intestinal populations of bacteria (van den Bogaard and Stobberingh, 2000). The production of ESBLs by *Enterobacteriaceae*, specifically by *E. coli*, has caused a major concern in several countries, being frequently implicated in human infections. Previous reports have described ESBL-containing *E. coli* strains in healthy wild animals (Pinto et al., 2010; Guenther et al., 2011).

The common occurrence of antimicrobial resistance in wildlife has several implications such as: the potential to serve as an environmental reservoir and melting pot of bacterial resistance; the zoonotic potential of enteric bacteria; and the potencial problems of the medical treatment of wildlife. This review aims to summarize the current knowledge on ESBL-*E. coli* and VRE in wildlife, in turn underlining the need for more large scale research, in particular sentinel studies to monitor the impact of multiresistant bacteria on wildlife.

## PHYLOGENETIC HISTORY AND GENETIC STRUCTURE

The combination of mutation and horizontal transfer has created the overall phylogenetic structure of *E. coli* and enterococci, resulted to currently recognized four main phylogenetic groups for *E. coli *and different species for enterococci; and their respective lineages. Thus, an appreciation of mutation and horizontal transfer as important evolutionary processes within bacteria, in general, contributes in understanding of their roles, distribution and mechanistic modes of behavior (Johnson, 2002).

A multilocus enzyme electrophoresis (MLEE)-based phenogram using 38 enzymes (Selander et al., 1986; Goullet and Picard, 1989) identified four main phylogenetic groups (A, B1, B2, and D) and two accessory groups (C and E) in *E. coli *(Selander et al., 1987; Herzer et al., 1990; Tenaillon et al., 2010). These phylogenetic groups were recovered using the 1,878 genes of the *Escherichia *spp. core genome and the 2.6 million nucleotides of the *E. coli *chromosomal backbone (Touchon et al., 2009), which permitted a robust phylogeny to be developed; the first split in the *E. coli* phylogenetic history leads to one branch including the strains of group B2 and a subgroup within D that we called group F75 and another branch containing the rest of the species (Touchon et al., 2009). The remaining strains of group D then appeared from this second branch, followed by group E (Tenaillon et al., 2010). Finally, groups A and B1 are sister groups whereas group B2 is included in an ancestral branch (Jaureguy et al., 2008; Touchon et al., 2009). The B2 group reveals the highest diversity at both the nucleotide and the gene content level (Touchon et al., 2009), supporting its early occurrence in the species lineage and suggesting that it has subspecies status (Lescat et al., 2009).

Clermont et al. (2000) described a triplex polymerase chain reaction (PCR) strategy to assign *E. coli* isolates quickly to one of these phylogroups. It sought three phylogenetic group markers, the *chu*A and *yja*A genes encoding hypothetical proteins and the TSPE4.C2 DNA sequences situated within a gene encoding putative lipase esterase, and groups were assigned based on different combinations of presence and/or absence of the three amplicons (Clermont et al., 2000).

Johnson et al. (2001) found that strains from phylogenetic groups B2 and D contained more virulence factors than strains from the phylogenetic groups A and B1. Usually, the extraintestinal pathogenic strains belong to groups B2 and D (Picard et al., 1999; Johnson and Stell, 2000), the commensal strains to groups A and B1 (Bingen et al., 1998), whereas the intestinal pathogenic strains belong to groups A, B1, and D (Pupo et al., 1997).

Nowadays, these phylogenetic groups differ in their ecological niches, life-history (Gordon and Cowling, 2003) and some characteristics, such as their ability to exploit different sugar sources, their antimicrobial resistance profiles and their growth rate (Carlos et al., 2010). A recent survey (Walk et al., 2007) demonstrated that the majority of the *E. coli* strains that are able to persist in the environment belong to the B1 phylogenetic group (Carlos et al., 2010).

Various researchers analyzed the distribution of the main phylogenetic groups among *E. coli *strains isolated from human and animal faces; it was found that the relative abundance of phylogenetic groups among mammals is dependent on the host diet, body mass, and climate (Gordon and Cowling, 2003; Carlos et al., 2010). A study analyzing fecal strains isolated from birds, non-human mammals, and humans, observed the prevalence of groups D and B1 in birds, A and B1 in non-human mammals, and A and B2 in humans. These different reports concluded that one of the main forces that shapes the genetic structure of *E. coli* populations among the hosts is domestication (Escobar-Paramo et al., 2006). Furthermore, other study in the south of French Guiana, human strains very rarely were observed as belonging to B2 phylogroup (3.7%) whereas wild animal strains were characterized by 46.1% belonging to B2 phylogroup (Lescat et al., 2013). Moreover, feces from zoo animals were analyzed and a prevalence of group B1 in herbivorous animals and a prevalence of group A in carnivorous and omnivorous animals were found (Baldy-Chudzik et al., 2008). Furthermore, domesticated animals have a decreased proportion of B2 strains than wild animals (from 30% in wild animals to 14 and 11% in farm and zoo animals, respectively) and an increased proportion of A strains (from 14% in wild animals to 27 and 26% in farm and zoo animals, respectively; Tenaillon et al., 2010).

**FIGURE 1:**
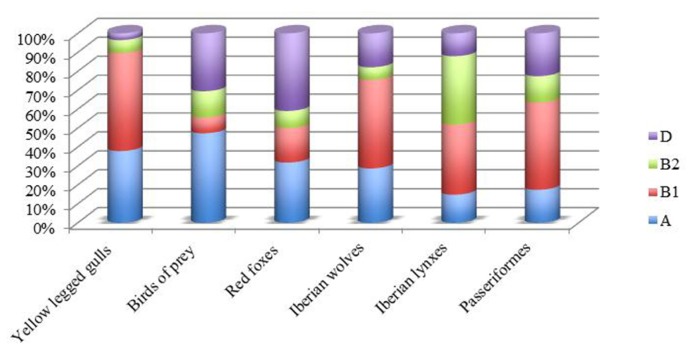
**Phylogenetic group distributions of *E. coli *in wild animal from Portugal**.

In Portugal, the prevalence of *E. coli* of groups A and B1 was observed in wild birds as seagulls (Radhouani et al., 2009), birds of prey (Radhouani et al., 2012b), and Passeriformes (Santos et al., 2013), but also in wild mammals as Iberian lynxes (Gonçalves et al., 2012a), red foxes (Radhouani et al., 2013), and in Iberian wolf (Gonçalves et al., 2013a; **Figure 1**). In addition, a report conducted by Simões et al. (2010) shows that 37% of all ESBL-*E. coli* isolated from seagulls belong to B2 or D phylogroup, a higher rate than previously reported (27% of all *E. coli*; Poeta et al., 2008).

It is interesting to note that fecal samples from red foxes showed that the *E. coli* isolates from phylogenetic groups A and D were predominant. Similar results were observed in chickens and swine (Machado et al., 2008), in wild boars (Poeta et al., 2009) and in wild birds (Radhouani et al., 2010a,b) in the same geographical area.

With the exception of *E*. *faecium *and *E*. *faecalis*, the enterococci are infrequently described to be involved in human pathogenesis (Jett et al., 1994; Devriese and Pot, 1995). Though, in some countries association of strains of *E*. *faecalis *and *E*. *faecium *with human disease has reached proportions of serious concern (Jett et al., 1994; Leclercq, 1997; Fisher and Phillips, 2009). However, these enterococci species can show significant differences in the incidence of virulence factors and antimicrobial resistance. Generally, *E. faecalis* appears to harbor more virulence traits while *E. faecium* strains were generally free of virulence factors (Eaton and Gasson, 2001). In addition, considering the distribution of the antimicrobial resistance according to the species, the *E. faecium* possessed a higher level of resistance than *E. faecalis* (Gin and Zhanel, 1996; Franz et al., 2001). Actually, the majority of hospital-derived isolates of *E. faecalis* cluster in three clonal complexes, CC2, CC9, and CC87, and included the highest proportion of multiresistant isolates. (Ruiz-Garbajosa et al., 2006; van Schaik and Willems, 2010; Weng et al., 2013), while the CC17 is a major group of genetic lineage of *E. faecium* that has widely spread worldwide and it is associated with hospital outbreaks (Willems et al., 2005; Willems and van Schaik, 2009). Although *E. faecalis* CC2 and CC9 strains has been detected outside hospitals in farm animals and environment (Freitas et al., 2011; Novais et al., 2013), to our knowledge has not been identified in wild animals. On the other hand, *E. faecium* CC17 was recovered from seagulls in Portugal (Radhouani et al., 2010c).

**FIGURE 2:**
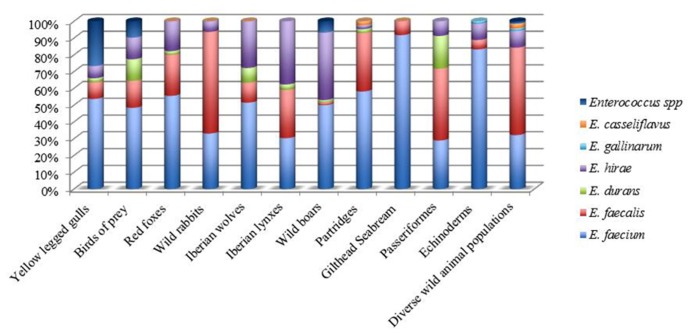
**Distribution of *Enterococcus* species in wild animals from Portugal**.

Recent comparisons of available genome sequences support the concept of a hospital-associated clade that is genetically distinct from most commensal isolates from animals and humans (Willems and van Schaik, 2009; van Schaik and Willems, 2010; Werner et al., 2011; Galloway-Pena et al., 2012). In Portugal, concerning wild animals, *E. faecium* were found to be the main species in seagulls (Radhouani et al., 2011b); birds of prey (Radhouani et al., 2012b), partridges (Silva et al., 2011b), Iberian wolf (Gonçalves et al., 2013a), red foxes (Radhouani et al., 2013), wild boars (Poeta et al., 2007a), gilthead seabream (Barros et al., 2011), and Echinoderms (Marinho et al., 2013), while *E. faecalis* was dominant in Passeriformes (Santos et al., 2013) and wild rabbits (Silva et al., 2010). On the other hand, *E. hirae* was the predominant specie isolated from Iberian lynx (Gonçalves et al., 2013b,c; **Figure 2**).

Along with seagulls and birds of prey in Portugal, migratory Canada geese report showed also the occurrence of *E. faecium* and *E. faecalis* species. Furthermore, different reports found that all enterococci isolates detected in wild mammals (Mallon et al., 2002) and also in glaucous gulls (Drobni et al., 2009) were *E. faecium* species. Another study performed in Brazil, showed the occurrence of *E. faecalis* in non-human primate (capuchin monkeys and common marmoset) fecal samples (Xavier et al., 2010). In American bison fecal samples, *E. casseliflavus* was the predominant species recovered with 62.4%, followed by *E. faecalis *(16%; Anderson et al., 2008).

With the arrival of next-generation sequencing (Mardis, 2008), it was quickly possible to investigate hundreds of strains to get a better knowledge at the whole-genome level the evolutionary processes acting in populations (Liti et al., 2009; MacLean et al., 2009; Schacherer et al., 2009), opening the era of “population genomics” (Tenaillon et al., 2010). Certainly further studies using new high throughput technologies are mandatory to completely understand the evolution of predominant clones and species in different hosts and environments (Santagati et al., 2012).

## DIVERSITY AND COMPLEXITY OF BACTERIAL NICHES

### VIRULENCE FACTORS

Large-scale epidemiological studies provide insight into the diversity and complexity of *E. coli* and enterococci niches (Tenaillon et al., 2010; Arias and Murray, 2012). Thus, genome plasticity has contributed to the emergence of new virulence traits: some clusters of genes or genomic islands, including pathogenicity islands (PAIs) should be discovered only in a subset of strains and favored in some specific environments. Furthermore, several alternative combinations of genes could promote similar adaptations to a given environment (Tenaillon et al., 2010). Gene gain and loss make fundamental contributions to new habitat adaptation and the emergence of new lineages (Dagan and Martin, 2007). Strains related to hospital infections were found to have significantly larger overall average genome size than strains from non-hospitalized humans or animals groups (Lebreton et al., 2013). The prevalence of virulence factors genes is variable among commensal populations. On a global scale, the human microbiota is characterized by a higher prevalence of virulence genes than the microbiota of other organisms (Skurnik et al., 2008). In animals, the presence of virulence genes increases with body mass, which reveals the gut complexity of larger animal (Skurnik et al., 2008). Thus, virulence factors and their change in prevalence among hosts may reflect some local adaptation to commensal habitats rather than virulence *per se *(Tenaillon et al., 2010).

### INTRA-SPECIES INTERACTIONS

Interactions between community members are required for community development and maintenance; and can also drive some diversification. Many genes that are carried by mobile elements code for traits that are expressed outside of the cell. Such traits are involved in bacterial sociality, such as the production of public goods, which benefit a cell’s neighbors, or the production of bacteriocins, which harm a cell’s neighbors. To out-compete other clones, the production of colicins can represent a useful strategy in a structured environment (Chao and Levin, 1981). Colicins are the most expansively studied bacteriocins produced by *E. coli*. This could permit unadapted strains to colonize the gut and, hence, allow numerous clones to coexist in the long term. It may also promote diversification in a clone, as some strains may try to benefit from the production of the colicin but avoid paying the associated cost. However, the secretion of bacteriocins can be defined as a “spiteful” behavior in which are costly for the producer and cause harm to other members of the population (Rankin et al., 2011).

Horizontal gene transfer is a key step in the evolution of bacterial pathogens. Chromosomal structures such as PAIs have been shown to extensively contribute to the evolution of bacterial pathogens by providing dynamic changes of the bacterial genome composition leading to a bacterial evolution in quantum leaps. Different mechanisms have been proposed for transfer of PAIs across the species border including phage transfer, mobilization by conjugative transposons and plasmids (Schubert et al., 2009). The fact that these microorganisms are less virulent than others is not reassuring, since the acquirement of virulence genes by bacteria is possible (Leclercq, 1997). Further research is required to characterize molecular and cellular interactions between the host and enterococci which lead to intra-species genetic transfer and virulence factors in enterococci species (Giridhara Upadhyaya et al., 2009).

### ANTIMICROBIAL RESISTANCE

Antimicrobial resistance is a worldwide problem in human and veterinary medicine. Commonly, it is usual that the major risk factor for the increase of this situation is an extensive use of antimicrobials that leads to the dissemination of resistant bacteria and resistance genes in animals and humans (van den Bogaard and Stobberingh, 2000). The appearance of multiresistant bacteria of human and veterinary origin is probably accompanied by co-contamination of the environment apparently leading to a great health concern (Grobbel et al., 2007). As in the hospital environment, the agricultural use of antimicrobials agents selects for antibiotic resistance. These antimicrobial drugs from both hospital and agricultural sources can persist in soil and aquatic environments, and the selective pressure imposed by these compounds may affect the treatment of human diseases (Thiele-Bruhn, 2003; Segura et al., 2009). In addition, the prophylactic use of antibiotics in fish farms has led to a rise in the number of resistant bacteria (Cabello, 2006). However, the use of antibiotics by humans is not the only selective pressure for antibiotic resistance in natural microbial communities: compounds and conditions that occur in these communities may provide additional selection pressures. In fact, most antimicrobial agents are produced by strains of fungi and bacteria that occur naturally in all environments, including soil (Martin and Liras, 1989).

Bacteria may also acquire resistance determinants without direct exposure to an antimicrobial through horizontally mobile elements including conjugative plasmids, integrons, and transposons (Middleton and Ambrose, 2005). These mobile elements can simply transfer antimicrobial resistance genes from one bacterium to another (Coque et al., 2008). Antimicrobial agents exert a selective pressure not only on pathogenic, but also on commensal bacteria of the intestinal tract of humans and animals (van den Bogaard and Stobberingh, 2000). In a broader view, increasing evidence suggests that components such as integrons and their gene cassettes played significant roles in genome evolution and fluidity within the bacterial kingdom (Duriez et al., 2001; Davies and Davies, 2010).

In *E. coli*, the phylogenetic group A strains (Mammeri et al., 2009) and some group D strains (Deschamps et al., 2009) are especially permissive to the development of resistance to third-generation cephalosporins. Conversely, phylogenetic group B2 strains are less resistant than the remaining strains (Picard and Goullet, 1989; Johnson et al., 1991, 1994), regardless of the molecular mechanism implicated in the acquisition of resistance, and have a lower prevalence of integrons in commensal *E. coli *strains from both human (Skurnik et al., 2005) and animal hosts (Skurnik et al., 2006). This could reveal the relative decrease of phylogenetic group B2 strains in domesticated animals in which antimicrobials are used considerably (Tenaillon et al., 2010).

The class of beta-lactam antibiotics is among the most important groups of antimicrobial agents in human and veterinary medicine. Besides the first widely used antimicrobial substance penicillin, other members of this family have gained a similar importance over the last decades, namely the first- to fourth- generation cephalosporins and the beta-lactamase-inhibitors. All beta-lactams interfere with the final stage of peptidoglycan synthesis through acting on penicillin-binding proteins, thereby preventing the bacterial cell wall from forming. The most common resistance mechanism of *Enterobacteriaceae* spp. against beta-lactams is the inactivation of the antibiotic by breaking up the nitrogen-carbonyl bond in the beta-lactam ring (Walsh, 2003). The emergence and wide dissemination of ESBLs among clinical *E. coli* isolates in hospitals, has caused a major concern in several countries, being frequently implicated in human infections. These infections have a great impact on public health due to an increased incidence of treatment failure and severity of disease. ESBLs mainly include TEM, SHV, and CTX-M enzymes. Among them, the highest number of variants described in the last years corresponds to the CTX-M family (Naas et al., 2007). The presence of CTX-M enzymes render *E. coli* resistant to a variety of beta-lactams, and are transferred via plasmids that can also include resistance genes to several unrelated classes of antimicrobial agents (Canton et al., 2012).

Carbapenems such as imipenem or meropenem possess the most consistent activity against ESBL-producing *Enterobacteriaceae *strains. Both antibiotics are considered the agents of choice in the treatment of infections due to ESBL-producing organisms (Nordmann et al., 2011) However, *Enterobacteriaceae*-producing carbapenemases have rapidly emerged and disseminated worldwide, including in the wild (Fischer et al., 2013). The carbapenemases, such as the New Delhi metallo-beta-lactamase 1 (NDM-1) hydrolyze all β-lactam antibiotics, including carbapenems, and their high potential for rapid, wide dissemination constitutes a major clinical and public health threat (Nordmann et al., 2009; Nordmann et al., 2011).

The increase in the occurrence of nosocomial infections caused by enterococci in particular *E. faecium*, is at least partly due to the wide variety of intrinsic and acquired resistances to glycopeptides and aminoglycosides, among others, posing a challenge to therapeutic options. Moreover, infections caused by other *Enterococcus *species (*E. faecalis*, *E. durans*, *E. mundtii*, *E. avium*, *E. raffinosus*, *E. gallinarum*, and *E. casseliflavus*) occasionally occur and warrant attention (Murray, 1990; Prakash et al., 2005). Enterococci are expert in acquiring and transferring elements that confer resistance to antimicrobials and they are also known to be intrinsically resistant to numerous antimicrobials. As a result, therapeutic alternatives for treatment of enterococcal infections are increasingly limited (Murray, 1990). Evolution of enterococci toward resistance to multiple antimicrobials is also a major cause of concern.

The acquisition of vancomycin resistance by enterococci has seriously affected the treatment and infection control of these organisms. Different types of glycopeptide resistance and their biochemical mechanisms have been described in enterococci: acquired type (*vanA, vanB*, *vanD*, *vanE*, *vanG*, and *vanL*); and low-level intrinsic type (*vanC*, associated with the *E. gallinarum*, *E. casseliflavus*, and *E. flavescens* species). VanA-type resistance, which was the first to be elucidated and which is the most common, is characterized by high levels of resistance to glycopeptides, vancomycin, and teicoplanin and is mediated by transposon Tn*1546* or closely related elements that are chromosomally or plasmid located (Arthur et al., 1996). The development of newer antimicrobial drugs, such as linezolid and daptomycin with activity against many VRE strains (Lee et al., 2007). has improved this situation; however resistance to these agents has already been described (Miller et al., 2013; Patel et al., 2013).

## WILDLIFE AS RESERVOIRS OF ANTIBIOTIC RESISTANCE

An important difficulty in evaluating the causal relationship between antimicrobial use and resistance is the confounding influence of geography: the co-localization of resistant bacterial species with antimicrobial use does not essentially involve causation and could represent the presence of environmental conditions and factors that have independently contributed to the incidence of resistance (Singer et al., 2006).

The collection of all antimicrobial resistance genes and their precursors in pathogenic and non-pathogenic bacteria and also in antimicrobial producing-organisms is referred as the antimicrobial resistome, a concept that has been advanced to serve as a framework for understanding the ecology of resistance on a global scale (Wright, 2010).

Usually, wildlife is not exposed to clinical antimicrobial agents but can acquire antimicrobial-resistant bacteria through contact with humans, animals and the environment, where water polluted with feces appears to be the most significant vector of contamination. The incidence of commensal and pathogenic bacteria in fecal contaminations can be expected to be a connection between the environment and settings with regular or even constant antimicrobial pressure (aquaculture, livestock farming, human, and veterinary clinical settings), resulting in a constant release of antimicrobial-resistant human and animal bacteria into the environment through wastewater or manure (Martinez, 2009). Additionally, the detection of antimicrobial-resistant bacteria in aquatic environments affected by human and animal wastewater and soil provides evidence for this hypothesis (Kummerer and Henninger, 2003). In this context the common use of antimicrobials in aquaculture is also of utmost importance due to possible direct influences on wild animals (Smith, 2008). As intestinal bacteria like *E. coli *and enterococci can be easily disseminated in different ecosystems through water, they are intensively used as indicator species for fecal pollution (Guenther et al., 2011). In this sense, it is essential to interpret the evolutionary and ecological forces that influence in the population structure of the commensal strains to fully understand the antimicrobial resistance and virulence of pathogenic strains. Certainly, the selective pressures in the habitats of commensal strains may coincidentally promote the emergence of antimicrobial resistance and virulence factors, rendering commensal strains reservoirs of virulent and resistant strains (Tenaillon et al., 2010).

Despite the commensal character of *E. coli *and enterococci, they are commonly involved in animal and human infections that implicate the use of antimicrobials, which increases public health preoccupations to the list of implications that arise from the spread of ESBL-*E. coli* and VRE into wildlife. Furthermore, the increasing frequency of community-acquired ESBL-*E. coli *and VRE infections and the occurrence in livestock farming has been observed recently, suggesting a successful transmission as well as persistence of ESBL-*E. coli *and VRE strains outside hospital settings. An additional parallel global phenomenon is the spread of ESBL-*E. coli *and VRE into the environment beyond human and domesticated animal populations, and this appears to be directly induced by antimicrobial practice (Guenther et al., 2011). This might be a significant cause of the community-onset of ESBL-*E. coli *and VRE infections but can result (i) in an involvement of wildlife in ESBL-*E. coli *and VRE spread and transmission into fragile environmental niches, (ii) in subsequent colonization of wild animal populations which can turn into an infectious source or even a reservoir of ESBL-*E. coli *and VRE, (iii) in new putative infection cycles between wildlife, domesticated animals and humans, and (iv) in difficulties of wildlife medical treatment (Guenther et al., 2011).

Monitoring the prevalence of resistance in indicator bacteria such as fecal *E. coli* and enterococci in different populations (animals, patients and healthy humans) makes it possible to compare the prevalence of resistance and to detect transfer of resistant bacteria or resistance genes from animals to humans and *vice versa* (Martel et al., 2001). Just recently, there has been increasing interest in resistant bacteria and resistance genes isolated from wild animals (Allen et al., 2011).

The degree of colonization varies a lot between different animal species (Gordon and Cowling, 2003). Therefore, ESBL-*E. coli *and VRE prevalence is clearly influenced by sampling schemes, by geographic regions, by host spectrum of these bacteria and by the degree of synanthropic behavior shown by host species (Allen et al., 2010). It is important also to take in consideration the limitations that occur interpreting these results. For instance, ESBL-*E. coli* prevalence in different Portuguese geographical areas ranged from 0.5% in birds of the remote Azores islands in the Atlantic Ocean (Silva et al., 2011a) to 32% for birds of the Portugal’s Northern Portuguese coast (Simões et al., 2010). A lower prevalence of ESBL-*E. coli* was also observed (0.8%) in glaucous-winged gulls of Kamchatka peninsula in Russia (Hernandez et al., 2010). These findings suggest that wild animals living in urban areas are more susceptible to carry ESBL-*E. coli *than those living in remote areas.

Due to their diversity in ecological niches and their ease in picking up human and environmental bacteria, wild birds might act as mirrors of human activities. Within the heterogeneous class of birds, two groups seem to be in the focus of ESBL-*E. coli* and VRE carriage in wildlife: birds of prey (Costa et al., 2006; Pinto et al., 2010; Radhouani et al., 2010a,b; Silva et al., 2011a) and waterfowl/water related species (Poeta et al., 2008; Bonnedahl et al., 2009; Dolejska et al., 2009; Guenther et al., 2010a; Hernandez et al., 2010; Literak et al., 2010a; Radhouani et al., 2010c; Simões et al., 2010; Garmyn et al., 2011). However, recent studies in Passeriformes have also described a significant incidence of VRE (Silva et al., 2011a, 2012; Oravcova et al., 2013a,b).

Although wild birds, such as birds of prey, have only rare contact with antimicrobial agents, in disagreement with the existence of direct selective pressure, they can be contaminated or colonized by resistant bacteria. Water contact and acquisition via food seem to be major aspects of transmission of resistant bacteria of human or veterinary origin to wild animals (Cole et al., 2005). In the other hand, wild birds such as seagulls are often opportunistic marine feeders along the shoreline or offshore, but also eat the food sources provided by humans, especially garbage. Migrating birds that travel long distance seem to act as transporters, or as reservoirs, of resistant bacteria and may consequently have a significant epidemiological role in the dissemination of resistance, as well as being mirrors of the spectrum of pathogenic microorganisms present in humans (Radhouani et al., 2010c; Silva et al., 2011a). Reports on marine fish showed the presence of ESBL-*E. coli *(Sousa et al., 2011) and VRE (Barros et al., 2012) in gilthead seabream, indicating a dissemination of ESBL-*E. coli* and VRE into the Atlantic ocean. Moreover, it has previously been demonstrated that seagulls shared strains with isolates cultured from wastewater treatment plants and landfills (Nelson et al., 2008). This highlights the possibility of bacterial exchange between human sewage and birds.

Another important host of these bacteria appears to be in wild rodents. Although these animals have previous been in the focus of research on ESBL in wildlife in different continents (Gilliver et al., 1999; Kozak et al., 2009; Guenther et al., 2010b; Literak et al., 2010b; Allen et al., 2011), they have only been detected in urban rats (Guenther et al., 2010a; Ho et al., 2011). On the other hand, VRE have been earlier described in wild rodents (Mallon et al., 2002). This synantropic species can easily pickup human waste and frequently interacts with human feces in the sewage system in urban environments and can therefore easily acquire multiresistant bacteria. Remarkably, wild boars have also been described as hosts of these bacteria in Europe, which might expose their omnivorous feeding behavior (Poeta et al., 2007b, 2009; Literak et al., 2010b). Recent studies revealed the presence of ESBL-producing *E. coli *(Gonçalves et al., 2012a,b) and VRE isolates (Gonçalves et al., 2011) in Iberian wolf and/or Iberian lynx. The incidence of ESBL-*E. coli *(Radhouani et al., 2012a) and VRE (Radhouani et al., 2011a) in red foxes may be due their diet as these wild animals usually hunt wild rabbits, small rodents and birds. It is important to point out that some studies reported the presence of antimicrobial-resistant isolates in wild rabbits (Figueiredo et al., 2009; Silva et al., 2010) and wild rodents (Kozak et al., 2009; Guenther et al., 2010b). Foxes are on top of the food chain, perhaps accumulating multiresistant bacteria from their prey (Grobbel et al., 2012). All these evidences may contribute in the acquisition and spread of antimicrobial-resistant bacteria even in the absence of direct antimicrobial pressure.

These wild animals act as reservoirs of resistance genes and they could spread resistant bacteria throughout the wild environment. These researchers address the issue of antimicrobial-resistant microorganism proliferation in the environment and the related potential human health and environmental impact.

The level of resistant bacteria detected in wild animals seems to relate well with the degree of association with human activity (Skurnik et al., 2006). In fact, human density, natural preservation state, livestock or the reserve of an area may be significant criteria for the proliferation of antimicrobial-resistant bacteria (Allen et al., 2010). Nevertheless, several studies report the occurrence of multidrug-resistant bacteria in remote places or preservation areas therefore underlining the complexity of the spread of antimicrobial resistance in wild animals. These discoveries propose, on one hand, an influence of migratory behavior of wild birds into remote areas, or on the other hand the omnipresence of human influence in various ecological niches of the planet via human feces (Guenther et al., 2011). Different reports showed that areas with high livestock and human density and an assumable frequent interaction of wildlife with human influenced habitats of any kind (livestock farms, landfills, sewage systems, or wastewater treatment facilities) result in a higher risk for wildlife to acquire antimicrobial-resistant bacteria (Allen et al., 2010).

## CONCLUSION

The strength of trillions upon trillions of microorganisms, combined with the ancient force of evolution by constant, insistent variation, will inevitably overpower the drugs. Their spectrum is selected to involve pathogenic bacteria and antimicrobials constantly select naturally resistant bacteria (American Academy of Microbiology, 2009). As bacteria quickly evolve to acquire resistance to the available antimicrobials, it is a constant race for scientists to develop effective strategies to combat infection and to reveal new therapeutic targets (Davies and Davies, 2010).

Moreover, antimicrobial resistance evolving and spreading among bacterial pathogens is a public health problem of increasing magnitude. Since the beginning of the antimicrobial era, the selective pressure caused by the use of antimicrobials in clinical, veterinary, husbandry, and agricultural practices is considered the major factor responsible for the occurrence and spread of antimicrobial-resistant bacteria. The evolution of antimicrobial resistance in bacteria is related to the evolution of antimicrobial production. Though, resistance has also been discovered in the absence of antimicrobial exposure, as in bacteria from wildlife, raising an interest about the mechanisms of emergence and persistence of resistant strains under similar conditions, and the implications for resistance control strategies (Pallecchi et al., 2008). Monitoring antimicrobial resistance in wildlife from remote areas could also be a useful tool to evaluate the impact of anthropic pressure (Thaller et al., 2010).

Singer et al. (2006) support that in ecological studies of antimicrobial resistance, there has possibly been too much focus on resistant organisms and not enough on resistance genes. Due to the capability of bacteria to transfer resistance genes, even among distantly related bacteria, analyses of antimicrobial resistance emergence, dissemination and persistence might be better conducted at the gene level (Singer et al., 2006).

Until now, genomics-based investigation into *E. coli* and enterococci has focused on the identification of genes directly implicated in virulence. However, the fundamental physiology and response mechanisms to environmental conditions of *E. coli* and enterococci remained relatively poorly understood. This is a serious oversight because during infection the microbial fitness is an important reason in the success of any microbial pathogen. Further genome-wide reports aiming to define genes that are important during infection and colonization or exposure to antimicrobials will deliver significant data on relevant aspects of *E. coli* and enterococcal biology. This knowledge can consequently be useful for the development of novel treatment approaches to combat microbial infections.

## Conflict of Interest Statement

The authors declare that the research was conducted in the absence of any commercial or financial relationships that could be construed as a potential conflict of interest.
